# International Work Group (IWG): Alignment Across Criteria

**DOI:** 10.1002/alz70856_107829

**Published:** 2026-01-09

**Authors:** Giovanni Frisoni

**Affiliations:** ^1^ University Hospitals and University of Geneva, Geneva, Switzerland

## Abstract

**Background:**

The IWG and AA diagnostic criteria are major references in the Alzheimer's domain. Although they share analogies, some key differences should be recognized.

**Method:**

The original IWG 2024 JAMA Neurology and AA 2024 Alzheimers Dement papers will be used as the major data references.

**Result:**

I will outline the similarities and differences between the IWG and the AA criteria for Alzheimer's disease from the point of view of the former. I will discuss similarities regarding: Aim of clinical research on AD and other cognitive disorders, Role of co‐pathology, Role of brain reserve, Biomarker use, Indication for anti‐β‐amyloid, Contra‐indication for anti‐β‐amyloid, and Health care delivery model. I will discuss differences regarding: Core question, Scientific discourse, Knowledge source, Definition of AD, Diagnosis of AD, and Interventions for AD.

**Conclusion:**

The differences between the two sets of criteria are not “just semantics”. A unified definition of AD is essential to advance the field and improve patient care.

